# Supplementary material for: A viral expression factor behaves as a prion
Source: Nat Commun. 2019 Jan 21;10:359. doi: 10.1038/s41467-018-08180-z (PMC6341119; doi:10.1038/s41467-018-08180-z)
Supplement: Supplementary file 1 — Supplementary Information [file 41467_2018_8180_MOESM1_ESM.pdf]

Supplementary Information

A viral expression factor behaves as a prion

Nan et al.

This file includes:

Supplementary Figure 1-9

Supplementary Table 1-4

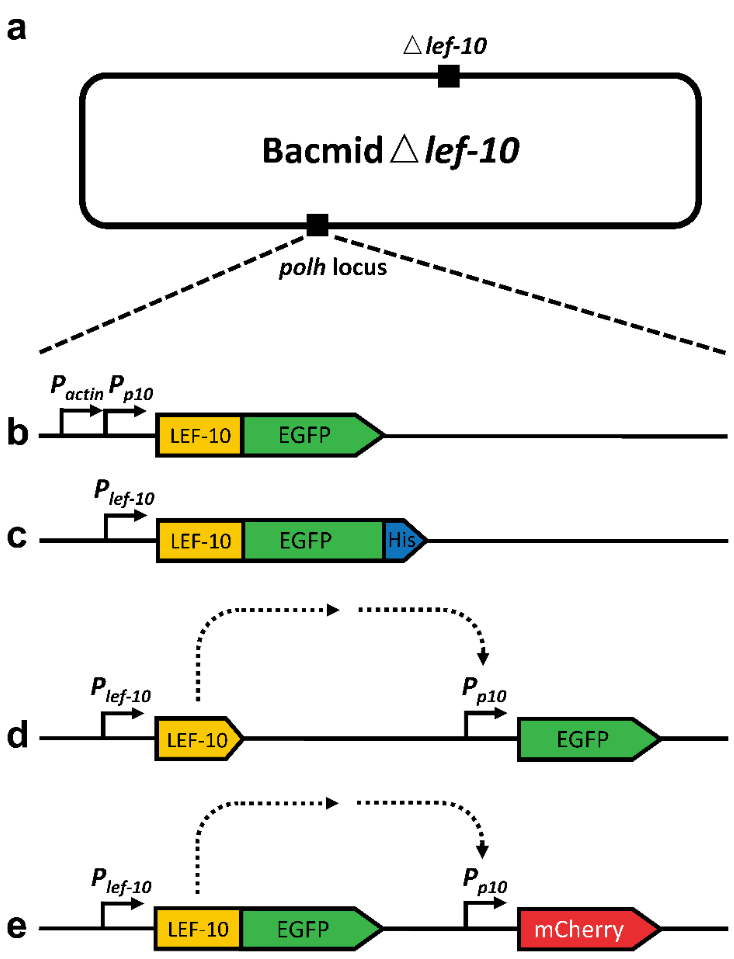

Supplementary Figure 1 Diagram of generation of recombinant baculoviruses based on the *lef-10* knock-out Bacmid via homologous recombination. Homologous recombination occurs between the BacmidΔ*lef-10* (**a**) and cassette fragments shown in **b**, **c**, **d** and **e**. **a** BacmidΔ*lef-10* comprises a *polh* locus as the recombination site[25]. **b** LEF-10-EGFP was regulated by two tandem promoters. LEF-10-EGFP gene driven by *chicken actin* promoter could rescue BacmidΔ*lef-10* as the promoter can drive the gene expression immediately after transfection or infection, and LEF-10-EGFP was over-expressed by the regulation of *p10* promoter (a strong promoter) in the late stage of infection. **c** LEF-10-EGFP gene driven by native *lef-10* promoter could rescue BacmidΔ*lef-10*. **d** LEF-10 gene driven by its native promoter could rescue BacmidΔ*lef-10*, and *p10* promoter driven EGFP could report the expression of baculovirus late gene. **e** LEF-10-EGFP gene driven by its native promoter could rescue BacmidΔ*lef-10*, and *p10* promoter driven mCherry could report the expression of baculovirus late gene.

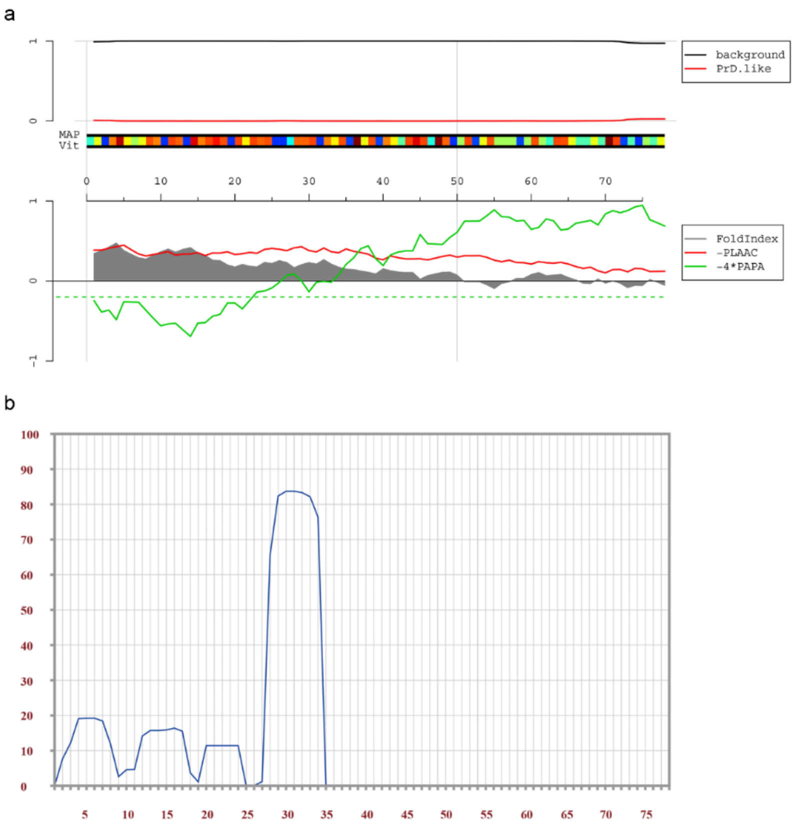

Supplementary Figure 2 Computational analysis of LEF-10. **a** PLAAC prediction tools show that LEF-10 does not contain a prion-like domain. **b** The TANGO algorithm predicts that LEF-10 contains four aggregating regions, including three low probability regions and one high probability region.

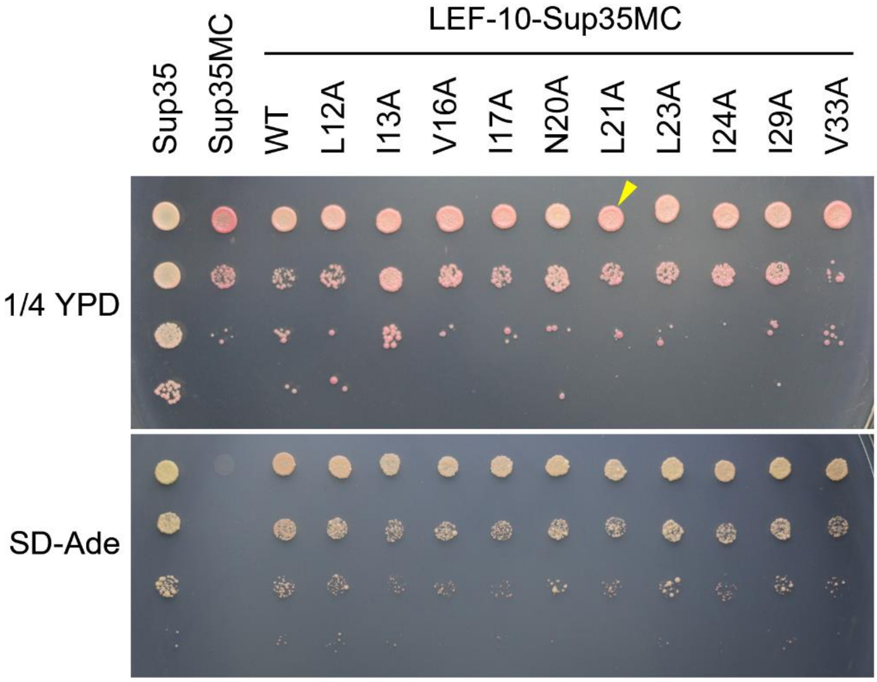

Supplementary Figure 3 Alanine substitution assays for ten highly conserved amino acid residues in LEF-10 C1 region. Alanine substitution assays were performed to assess the role of the ten highly conserved amino acid residues in maintaining the LEF-10 prion properties. The strains harboring LEF-10-Sup35MC mutants showed varying degree of color change, from white to faint red. All strains growing on SD-Ade medium indicated the read-through of *ade1-14* premature stop codon, and the result suggested that the single alanine substitutions of these conserved amino acid residues in LEF-10 could not fully abolish the prion prone of LEF-10.

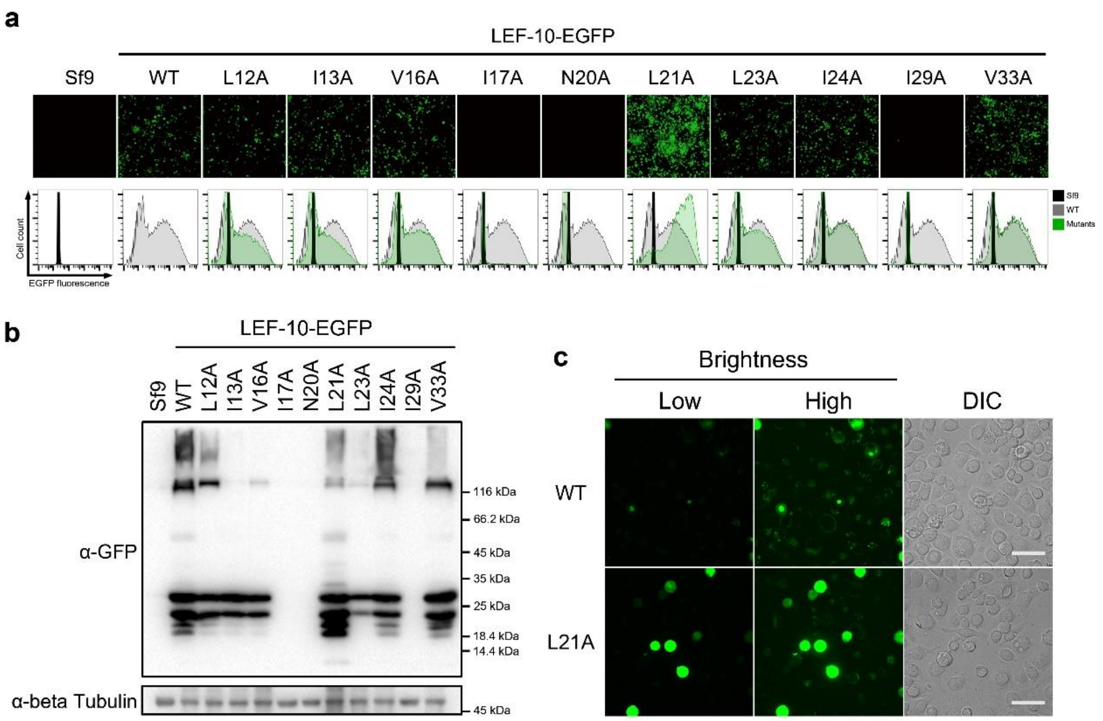

Supplementary Figure 4 Virus rescue assays using over-expressed wild-type LEF-10 and LEF-10 mutants. Wild-type LEF-10 and 10 LEF-10 mutants fused with EGFP were driven by a tandem *actin*/*p10* promoter (Supplementary Fig. 1b). **a** Baculovirus-infected *Sf*9 cells were observed under a fluorescence microscope (top panel) and analyzed by flow cytometry (bottom panel) at 48 hpi. Among the LEF-10 mutants, LEF-10^{L21A} displayed much higher activity than wild-type LEF-10, while three LEF-10 variants (LEF-10^{I17A}, LEF-10^{N20A} and LEF-10^{I29A}) failed to rescue viruses. Other LEF-10 mutants showed similar activity with wild-type LEF-10 in the virus rescue assay. Uninfected *Sf*9 cells were examined as control. **b** Western blot analysis of baculovirus-infected *Sf*9 cells expressing LEF-10-EGFP chimeras. The protein levels detected by an anti-GFP antibody were consistent with their corresponding fluorescence intensity (**a**). The protein level of beta-tubulin in the cell lysates was determined as a loading control. **c** Laser confocal microscopy images of over-expressed LEF-10-EGFP and LEF-10^{L21A}-EGFP in infected *Sf*9 cells at 48 hpi. Wild-type LEF-10-EGFP formed aggregates, whereas the LEF-10^{L21A}-EGFP mutant is evenly distributed in baculovirus-infected *Sf*9 cells with low brightness. Non-diffuse fluorescence exhibited by LEF-10^{L21A}-EGFP could be observed in some cells with high brightness. Scale bar, 50 μm.

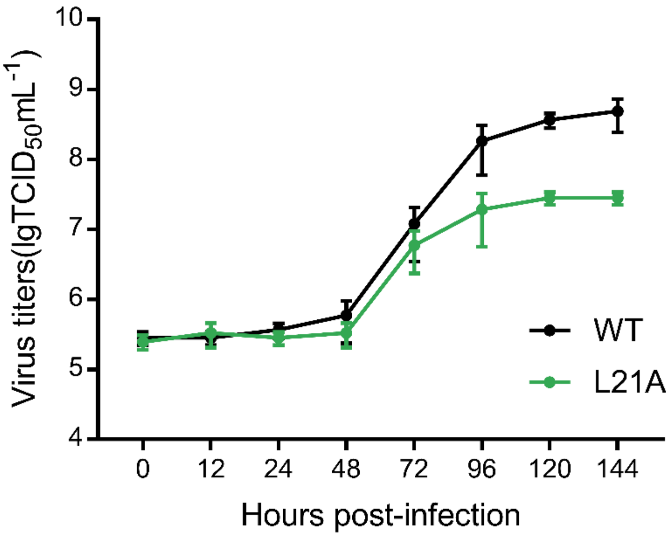

Supplementary Figure 5 One-step growth curve of two recombinant viruses expressing LEF-10 and LEF-10^{L21A}. *Sf*9 cells were infected with recombinant viruses expressing LEF-10 or LEF-10^{L21A} at an MOI of 0.5. The virus titers were determined by a $TCID_{50}$ endpoint dilution assay. The virus titers were converted to log base 10. $TCID_{50}$, 50% tissue culture infective dose. Data are represented as mean±SD from three replicates. Source data are provided as a Source Data file.

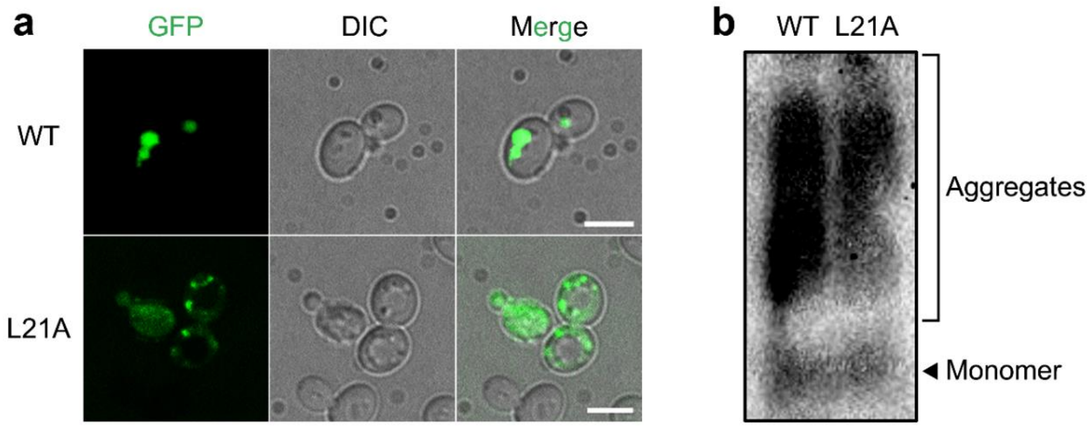

Supplementary Figure 6 Characterization of GFP-tagged LEF-10 and GFP-tagged LEF-10^{L21A} in yeast cells. **a** Laser confocal microscopy images displayed that LEF-10-GFP and LEF-10^{L21A}-GFP fusion proteins exhibit different non-diffuse florescence patterns in yeast cell after 4 hours expression induced by 25 μM $CuSO_4$. LEF-10-GFP could form foci, whereas the LEF-10^{L21A}-GFP formed punctate fluorescence pattern. Scale bar, 5 μm. **b** SDD-AGE analysis of LEF-10-GFP and LEF-10^{L21A}-GFP fusion proteins. The SDS-resistant aggregates could be detected in cell lysates of yeast strains containing LEF-10-GFP and LEF-10^{L21A}-GFP fusion proteins using α-GFP antibody.

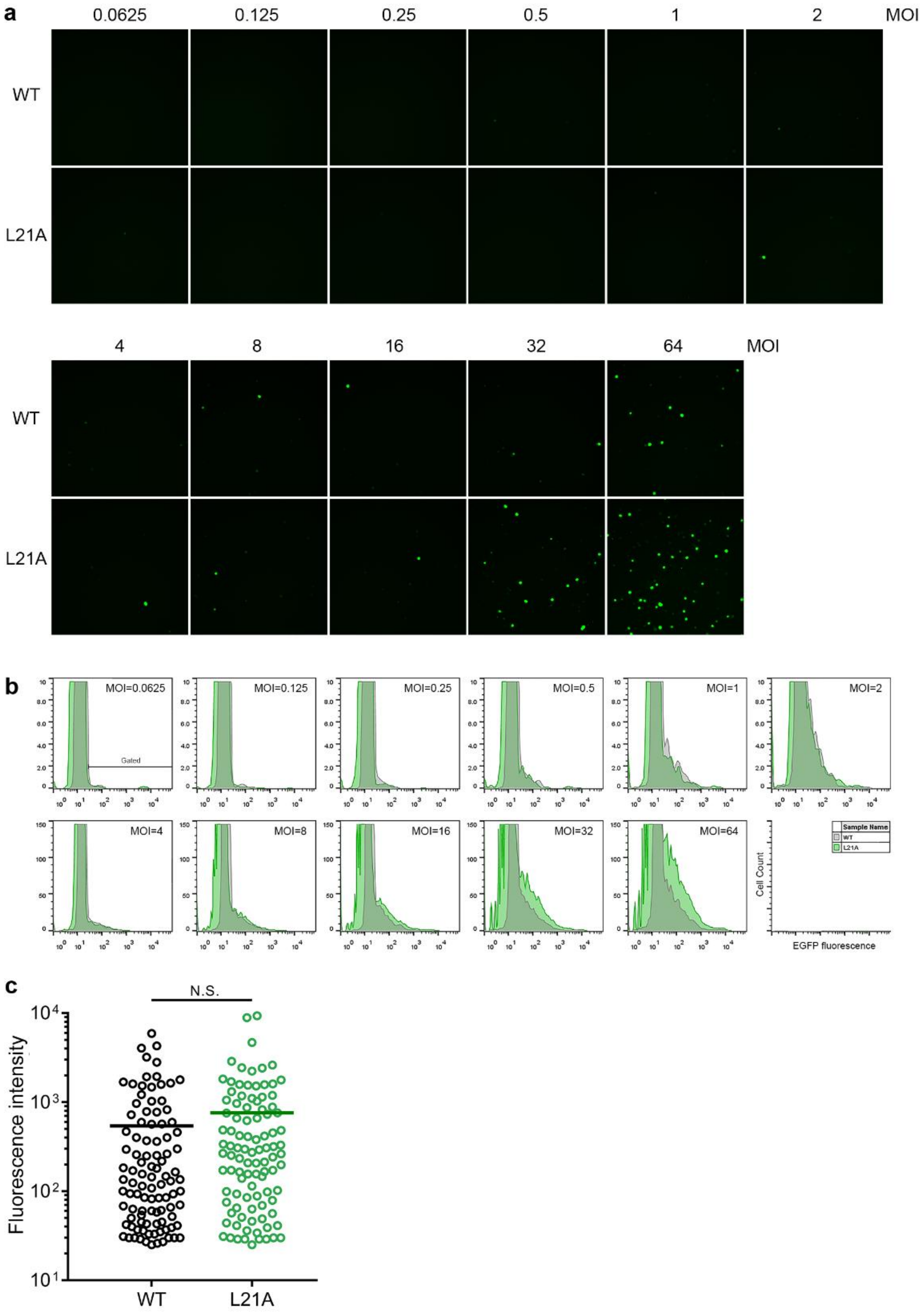

Supplementary Figure 7 Comparison of LEF-10-regulated late gene expression in infected *Sf*9 cells. *Sf*9 cells were infected with recombinant baculovirus encoding LEF-10 or LEF-10^{L21A} under the control of native *lef-10* promoter. EGFP driven by *p10* promoter was detected as a reporter of late gene expression (Supplementary Fig. 1d). The sensor system functions by transmitting the aggregation state of LEF-10 to downstream gene reporter

EGFP outputs. Two-fold dilutions of viruses at MOI of 0.0625, 0.125, 0.25, 0.5, 1.0, 2.0, 4.0, 8.0, 16.0, 32.0 and 64.0 were used for the *Sf*9 cell infection. Infected cells were imaged and collected at 36 hpi. **a** Fluorescence images showed that the baculovirus late gene expression regulated by LEF-10 was at comparable levels with that regulated by LEF-10^{L21A} at low MOIs (MOI≤8). However, following the protein expression increase at high MOIs, the late gene expression regulated by LEF-10 was significantly lower than the gene expression regulated by LEF-10^{L21A}. **b** Flow cytometry analysis confirmed that baculovirus late gene expression regulated by LEF-10 was close to the gene expression levels regulated by LEF-10^{L21A} at low MOIs (MOI≤8), but was much lower than that regulated by LEF-10^{L21A} at high MOIs. **c** Fluorescence intensity of *p10* promoter-driven EGFP in 100 infected cells. 100 infected *Sf*9 cells were gated (MOI≤1) and their EGFP fluorescence intensity was measured at 36 hpi using FlowJo® software. Bars represented mean. Two-tailed unpaired Student's *t*-test was performed (N.S., not significant). *P* value =0.2094.

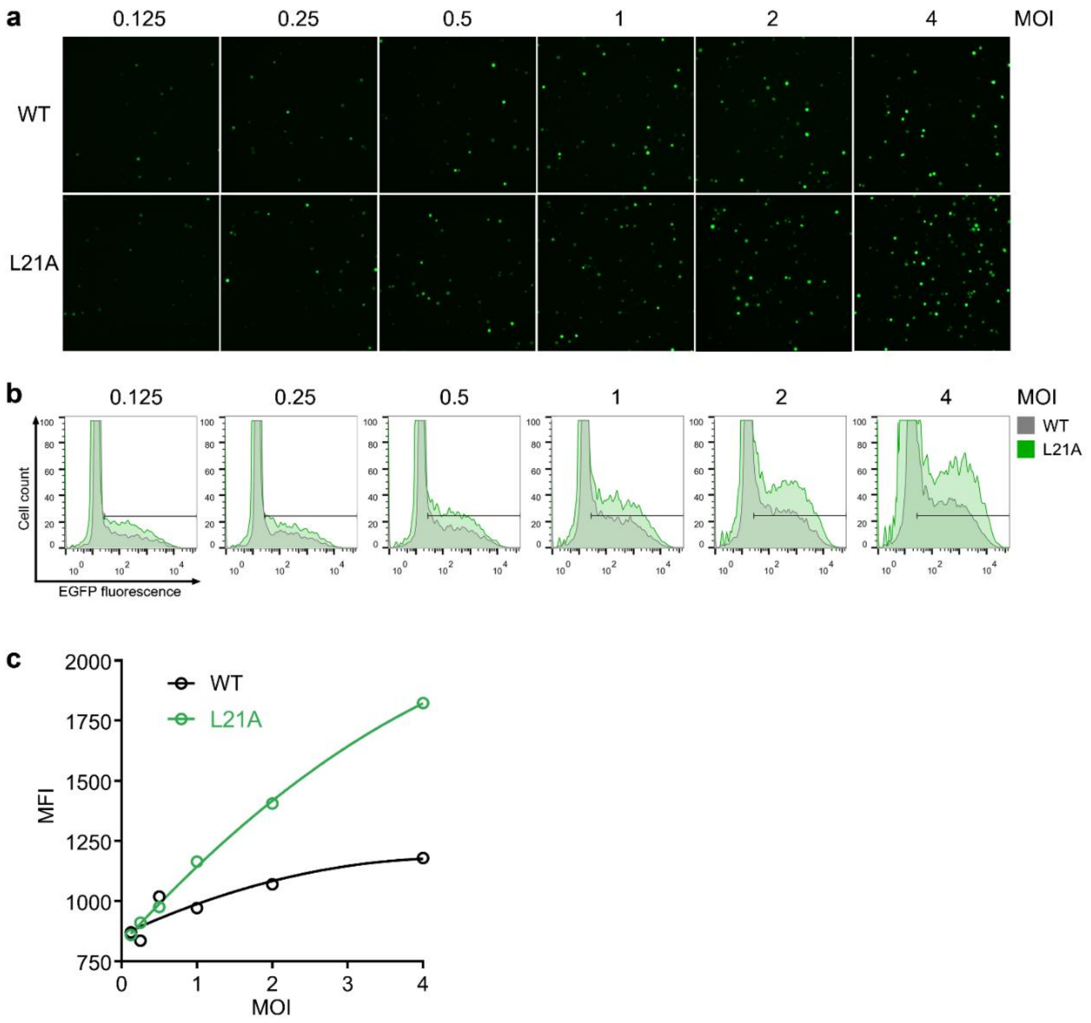

Supplementary Figure 8 Comparison of mean fluorescence intensity (MFI) in infected *Sf*9 cells. *Sf*9 cells were infected with recombinant baculovirus encoding LEF-10 or LEF-10^{L21A} under the control of the native *lef-10* promoter. EGFP driven by *p10* promoter was detected as a reporter of late gene expression (Supplementary Fig. 1d). Two-fold dilutions of viruses at MOI of 0.125, 0.25, 0.5, 1.0, 2.0 and 4.0 were used for the *Sf*9 cell infection. Images were captured by fluorescence microscope (**a**) and cell samples were collected and analyzed by flow cytometry (**b, c**), at 60 hpi. **a** Fluorescence images showed that the baculovirus late gene expression regulated by LEF-10 was at comparable levels with that regulated by LEF-10^{L21A} at low MOIs (MOI≤0.5). However, at high MOIs, the late gene expression regulated by LEF-10 was significantly lower than the gene expression regulated by

LEF-10^{L21A}. **b, c** Flow cytometry analysis confirmed that baculovirus late gene expression regulated by LEF-10 was close to the gene expression levels regulated by LEF-10^{L21A} at low MOIs, but was much lower than that regulated by LEF-10^{L21A} at high MOIs. Cells were infected in parallel with a serial dilutions of viruses expressing wild-type LEF-10 or the LEF-10^{L21A} mutant. In all samples, infected *Sf*9 cells were gated and the mean fluorescence intensity (MFI) was calculated by FlowJo® software. Source data are provided as a Source Data file.

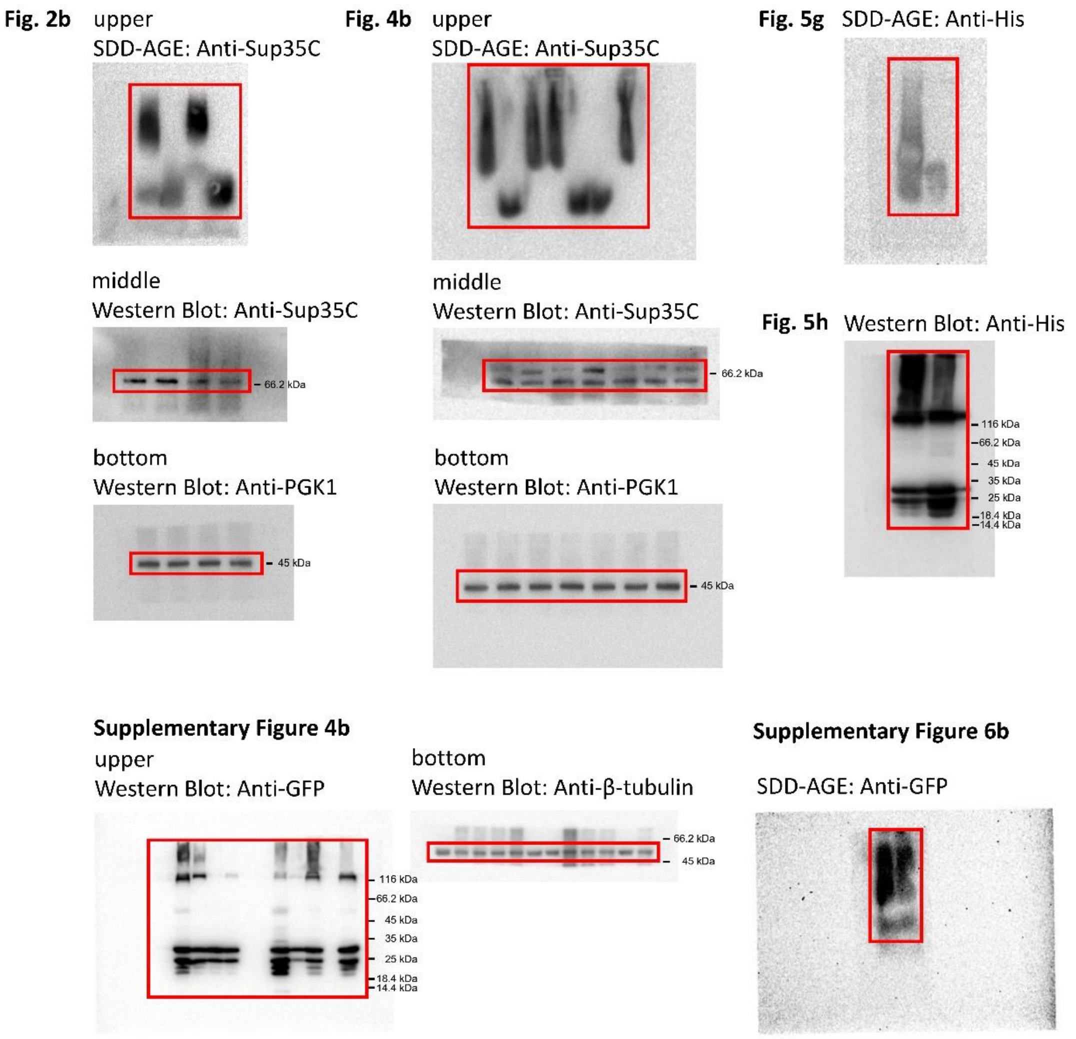

Supplementary Figure 9 Uncropped scans of immunoblotting results. Red boxes highlight lanes used in figures.

Supplementary Table 1 Plasmids used in this study.

Vectors Name	Plasmid Description	Source
pUKC1620	pRS313-P_{sup35}:Sup35	50
pUKC1620-△N	pRS313-P_{sup35}:Sup35MC	this study
pUKC1620-LEF-10	pRS313-P_{sup35}:LEF-10-Sup35MC	this study
pUKC1620-LEF-$10_{1\text{-}41}$	pRS313-P_{sup35}:LEF-$10_{1\text{-}41}$-Sup35MC	this study
pUKC1620-LEF-$10_{35\text{-}62}$	pRS313-P_{sup35}:LEF-$10_{35\text{-}62}$-Sup35MC	this study
pUKC1620-LEF-$10_{54\text{-}78}$	pRS313-P_{sup35}:LEF-$10_{54\text{-}78}$-Sup35MC	this study
pUKC1620-LEF-$10_{12\text{-}34}$	pRS313-P_{sup35}:LEF-$10_{12\text{-}34}$-Sup35MC	this study
pUKC1620-LEF-10^{L12A}	pRS313-P_{sup35}:LEF-10^{L12A}-Sup35MC	this study
pUKC1620-LEF-10^{I13A}	pRS313-P_{sup35}:LEF-10^{I13A}-Sup35MC	this study
pUKC1620-LEF-10^{V16A}	pRS313-P_{sup35}:LEF-10^{V16A}-Sup35MC	this study
pUKC1620-LEF-10^{I17A}	pRS313-P_{sup35}:LEF-10^{I17A}-Sup35MC	this study
pUKC1620-LEF-10^{N20A}	pRS313-P_{sup35}:LEF-10^{N20A}-Sup35MC	this study
pUKC1620-LEF-10^{L21A}	pRS313-P_{sup35}:LEF-10^{L21A}-Sup35MC	this study
pUKC1620-LEF-10^{L23A}	pRS313-P_{sup35}:LEF-10^{L23A}-Sup35MC	this study
pUKC1620-LEF-10^{I24A}	pRS313-P_{sup35}:LEF-10^{I24A}-Sup35MC	this study
pUKC1620-LEF-10^{I29A}	pRS313-P_{sup35}:LEF-10^{I29A}-Sup35MC	this study
pUKC1620-LEF-10^{V33A}	pRS313-P_{sup35}:LEF-10^{V33A}-Sup35MC	this study
p6431	pRS313-P_{CUP1}:GFP	S. L. L.
p6431-LEF-10	pRS313-P_{CUP1}:LEF-10-GFP	this study
p6431-LEF-10^{L21A}	pRS313-P_{CUP1}:LEF-10^{L21A}-GFP	this study
pTriEx-LEF-10-EGFP	pTriEx-$P_{actin\text{-}p10}$:LEF-10-EGFP	this study
pTriEx-LEF-10^{L12A}-EGFP	pTriEx-$P_{actin\text{-}p10}$:LEF-10^{L12A}-EGFP	this study
pTriEx-LEF-10^{I13A}-EGFP	pTriEx-$P_{actin\text{-}p10}$:LEF-10^{I13A}-EGFP	this study
pTriEx-LEF-10^{V16A}-EGFP	pTriEx-$P_{actin\text{-}p10}$:LEF-10^{V16A}-EGFP	this study
pTriEx-LEF-10^{I17A}-EGFP	pTriEx-$P_{actin\text{-}p10}$:LEF-10^{I17A}-EGFP	this study
pTriEx-LEF-10^{N20A}-EGFP	pTriEx-$P_{actin\text{-}p10}$:LEF-10^{N20A}-EGFP	this study
pTriEx-LEF-10^{L21A}-EGFP	pTriEx-$P_{actin\text{-}p10}$:LEF-10^{L21A}-EGFP	this study
pTriEx-LEF-10^{L23A}-EGFP	pTriEx-$P_{actin\text{-}p10}$:LEF-10^{L23A}-EGFP	this study
pTriEx-LEF-10^{I24A}-EGFP	pTriEx-$P_{actin\text{-}p10}$:LEF-10^{I24A}-EGFP	this study
pTriEx-LEF-10^{I29A}-EGFP	pTriEx-$P_{actin\text{-}p10}$:LEF-10^{I29A}-EGFP	this study
pTriEx-LEF-10^{V33A}-EGFP	pTriEx-$P_{actin\text{-}p10}$:LEF-10^{V33A}-EGFP	this study
pTriEx-$P_{lef\text{-}10}$-LEF-10-EGFP	pTriEx-$P_{lef\text{-}10}$-LEF-10-EGFP-His	25
pTriEx-$P_{lef\text{-}10}$-LEF-10^{L21A}-EGFP	pTriEx-$P_{lef\text{-}10}$-LEF-10^{L21A}-EGFP-His	this study
pTriEx-EGFP	pTriEx-P_{p10}:EGFP	this study
pTriEx-LEF-10/EGFP	pTriEx-$P_{lef\text{-}10}$:LEF-10/P_{p10}:EGFP	this study
pTriEx-LEF-10^{L21A}/EGFP	pTriEx-$P_{lef\text{-}10}$:LEF-10^{L21A}/P_{p10}:EGFP	this study
pTriEx-LEF-10-EGFP/mCherry	pTriEx-$P_{lef\text{-}10}$:LEF-10-EGFP/P_{p10}:mCherry	25
pTriEx-LEF-10^{L21A}-EGFP/mCherry	pTriEx-$P_{lef\text{-}10}$:LEF-10^{L21A}-EGFP/P_{p10}:mCherry	this study

Supplementary Table 2 *Saccharomyces cerevisiae* strains used in this study.

Strains Name	Genotype	Construction
LJ14	*MATa ade1-14 trp1-289 his3Δ-200 ura3-52 leu2-3,112*; *SUP35::loxP*; p[SUP35-URA3]; [PSI^+]	50
LJ14-SUP1	*MATa ade1-14 trp1-289 his3Δ-200 ura3-52 leu2-3,112*; *SUP35::loxP*; pUKC1620; [PSI^+]	[PSI^+]; plasmid shuffle using LJ14
LJ14-SUP2	*MATa ade1-14 trp1-289 his3Δ-200 ura3-52 leu2-3,112*; *SUP35::loxP*; pUKC1620; [psi^-]	[psi^-]
LJ14-SUP3	*MATa ade1-14 trp1-289 his3Δ-200 ura3-52 leu2-3,112*; *SUP35::loxP*; pUKC1620-△N;	plasmid shuffle using LJ14
LJ14-LEF1	*MATa ade1-14 trp1-289 his3Δ-200 ura3-52 leu2-3,112*; *SUP35::loxP*; pUKC1620-LEF-10; [PSI^+]-like	[PSI^+]-like; plasmid shuffle using LJ14
LJ14-LEF2	*MATa ade1-14 trp1-289 his3Δ-200 ura3-52 leu2-3,112*; *SUP35::loxP*; pUKC1620-LEF-10; [psi^-]-like	[psi^-]-like; plasmid shuffle using LJ14
LJ14-LEF3	*MATa ade1-14 trp1-289 his3Δ-200 ura3-52 leu2-3,112*; *SUP35::loxP*; pUKC1620-LEF-$10_{1\text{-}41}$	plasmid shuffle using LJ14
LJ14-LEF4	*MATa ade1-14 trp1-289 his3Δ-200 ura3-52 leu2-3,112*; *SUP35::loxP*; pUKC1620-LEF-$10_{35\text{-}62}$	plasmid shuffle using LJ14
LJ14-LEF5	*MATa ade1-14 trp1-289 his3Δ-200 ura3-52 leu2-3,112*; *SUP35::loxP*; pUKC1620-LEF-$10_{54\text{-}78}$	plasmid shuffle using LJ14
LJ14-LEF6	*MATa ade1-14 trp1-289 his3Δ-200 ura3-52 leu2-3,112*; *SUP35::loxP*; pUKC1620-LEF-$10_{12\text{-}34}$	plasmid shuffle using LJ14
LJ14-LEF7	*MATa ade1-14 trp1-289 his3Δ-200 ura3-52 leu2-3,112*; *SUP35::loxP*; pUKC1620-LEF-10^{L12A}	plasmid shuffle using LJ14
LJ14-LEF8	*MATa ade1-14 trp1-289 his3Δ-200 ura3-52 leu2-3,112*; *SUP35::loxP*; pUKC1620-LEF-10^{I13A}	plasmid shuffle using LJ14
LJ14-LEF9	*MATa ade1-14 trp1-289 his3Δ-200 ura3-52 leu2-3,112*; *SUP35::loxP*; pUKC1620-LEF-10^{V16A}	plasmid shuffle using LJ14
LJ14-LEF10	*MATa ade1-14 trp1-289 his3Δ-200 ura3-52 leu2-3,112*; *SUP35::loxP*; pUKC1620-LEF-10^{I17A}	plasmid shuffle using LJ14
LJ14-LEF11	*MATa ade1-14 trp1-289 his3Δ-200 ura3-52 leu2-3,112*; *SUP35::loxP*; pUKC1620-LEF-10^{N20A}	plasmid shuffle using LJ14
LJ14-LEF12	*MATa ade1-14 trp1-289 his3Δ-200 ura3-52 leu2-3,112*; *SUP35::loxP*; pUKC1620-LEF-10^{L21A}	plasmid shuffle using LJ14
LJ14-LEF13	*MATa ade1-14 trp1-289 his3Δ-200 ura3-52 leu2-3,112*; *SUP35::loxP*; pUKC1620-LEF-10^{L23A}	plasmid shuffle using LJ14
LJ14-LEF14	*MATa ade1-14 trp1-289 his3Δ-200 ura3-52 leu2-3,112*; *SUP35::loxP*; pUKC1620-LEF-10^{I24A}	plasmid shuffle using LJ14
LJ14-LEF15	*MATa ade1-14 trp1-289 his3Δ-200 ura3-52 leu2-3,112*; *SUP35::loxP*; pUKC1620-LEF-10^{I29A}	plasmid shuffle using LJ14
LJ14-LEF16	*MATa ade1-14 trp1-289 his3Δ-200 ura3-52 leu2-3,112*; *SUP35::loxP*; pUKC1620-LEF-10^{V33A}	plasmid shuffle using LJ14

Supplementary Table 2 (count.)

Strains Name	Genotype	Construction
LJ14-LEF17	*MATa ade1-14 trp1-289 his3Δ-200 ura3-52 leu2-3,112*; *SUP35::loxP*; hsp104::ura3; pUKC1620	LJ14-SUP1 *hsp104::Ura3*
LJ14-LEF18	*MATa ade1-14 trp1-289 his3Δ-200 ura3-52 leu2-3,112*; *SUP35::loxP*; hsp104::ura3; pUKC1620-LEF-10	LJ14-LEF1 *hsp104::Ura3*
LJ14-LEF19	*MATa ade1-14 trp1-289 his3Δ-200 ura3-52 leu2-3,112*; *SUP35::loxP*; hsp104::ura3; pUKC1620-LEF-$10_{1\text{-}41}$	LJ14-LEF3 *hsp104::Ura3*
LJ14-LEF20	*MATa ade1-14 trp1-289 his3Δ-200 ura3-52 leu2-3,112*; *SUP35::loxP*; hsp104::ura3; pUKC1620-LEF-$10_{12\text{-}34}$	LJ14-LEF6 *hsp104::Ura3*
LJ14-LEF-10-GFP	*MATa ade1-14 trp1-289 his3Δ-200 ura3-52 leu2-3,112*; *SUP35::loxP*; pUKC1620; [PSI+]; p6431-LEF-10	LJ14-SUP1 transformed with p6431-LEF-10
LJ14-LEF-10^{L21A}-GFP	*MATa ade1-14 trp1-289 his3Δ-200 ura3-52 leu2-3,112*; *SUP35::loxP*; pUKC1620; [PSI+]; p6431-LEF-10^{L21A}	LJ14-SUP1 transformed with p6431-LEF-10^{L21A}

Supplementary Table 3 Viruses used in this study.

Virus Name	Target Encoding Proteins	Source
vAc/$P_{lef\text{-}10}$:LEF-10-EGFP-P_{p10}:mCherry	LEF-10-EGFP and mCherry as the late gene reporter	25
vAc/$P_{lef\text{-}10}$:LEF-10L21A-EGFP-P_{p10}:mCherry	LEF-10^{L21A}-EGFP and mCherry as the late gene reporter	this study
vAc/$P_{actin\text{-}p10}$:LEF-10-EGFP	over-expressed LEF-10-EGFP	this study
vAc/$P_{actin\text{-}p10}$:LEF-10^{L12A}-EGFP	over-expressed LEF-10^{L12A}-EGFP	this study
vAc/$P_{actin\text{-}p10}$:LEF-10^{I13A}-EGFP	over-expressed LEF-10^{I13A}-EGFP	this study
vAc/$P_{actin\text{-}p10}$:LEF-10^{V16A}-EGFP	over-expressed LEF-10^{V16A}-EGFP	this study
vAc/$P_{actin\text{-}p10}$:LEF-10^{I17A}-EGFP	over-expressed LEF-10^{I17A}-EGFP	this study
vAc/$P_{actin\text{-}p10}$:LEF-10^{N20A}-EGFP	over-expressed LEF-10^{N20A}-EGFP	this study
vAc/$P_{actin\text{-}p10}$:LEF-10^{L21A}-EGFP	over-expressed LEF-10^{L21A}-EGFP	this study
vAc/$P_{actin\text{-}p10}$:LEF-10^{L23A}-EGFP	over-expressed LEF-10^{L23A}-EGFP	this study
vAc/$P_{actin\text{-}p10}$:LEF-10^{I24A}-EGFP	over-expressed LEF-10^{I24A}-EGFP	this study
vAc/$P_{actin\text{-}p10}$:LEF-10^{I29A}-EGFP	over-expressed LEF-10^{I29A}-EGFP	this study
vAc/$P_{actin\text{-}p10}$:LEF-10^{V33A}-EGFP	over-expressed LEF-10^{V33A}-EGFP	this study
vAc/$P_{lef\text{-}10}$:LEF-10-EGFP	LEF-10-EGFP-His	25
vAc/$P_{lef\text{-}10}$:LEF-10^{L21A}-EGFP	LEF-10^{L21A}-EGFP-His	this study
vAc/$P_{lef\text{-}10}$:LEF-10-P_{p10}:EGFP	LEF-10 and EGFP as the late gene reporter	this study
vAc/$P_{lef\text{-}10}$:LEF-10^{L21A}-P_{p10}:EGFP	LEF-10^{L21A} and EGFP as the late gene reporter	this study

Supplementary Table 4 Primers used in this study.

Primer Name	Sequences (5'-3')
SUPMC-FA	tcaaactctagatctgaattcatgtctttgaacgact
SUPMC-FB	aacatctatatctgcccactagcaacaatgtcggattcaaactctagatctgaattc
SUPMC-FC	aacaggatcctcttcatcgacttgctcggaataacatctatatctgcccactag
SUPMC-RABC	gcgggagctctatattgagaggtgaag
LEF-10-1F	agcctctagaacgaacgtatggttc
LEF-10-35F	gccgtctagagaccaagaaaccgatcaag
LEF-10-54F	attatctagaaccgatgcggccgcccaag
LEF-10-41R	cggcgaattcaacttgatcggtttcttgg
LEF-10-62R	tacggaattctgcatcggcttgggcggc
LEF-10-78R	tagagaattccgtggacgcgttactttg
LEF-10-L12A_Fm	gcgacggacgtcaacgctatcaattgtgtactg
LEF-10-L12A_Rm	cagtacacaattgatagcgttgacgtccgtcg
LEF-10-I13A _Fm	acggacgtcaacctggctaattgtgtactgaa
LEF-10-I13A _Rm	ttcagtacacaattagccaggttgacgtccg
LEF-10-V16A _Fm	aacctgatcaattgtgctctgaaagataatttat
LEF-10-V16A _Rm	taaattatctttcagagcacaattgatcaggtt
LEF-10-L17A _Fm	ctgatcaattgtgtagctaaagataatttatt
LEF-10-L17A _Rm	aaataaattatctttagctacacaattgatcag
LEF-10-N20A _Fm	tgtgtactgaaagatgctttatttttgatagat
LEF-10-N20A _Rm	atctatcaaaaataaagcatctttcagtacaca
LEF-10-L21A _Fm	gtactgaaagataatgctttttgatagataat
LEF-10-L21A _Rm	attatctatcaaaaaagcattatctttcagtacac
LEF-10-L23A _Fm	aaagataatttatttgctatagataataattac
LEF-10-L23A _Rm	gtaattattatctatagcaaataaattatctttcag
LEF-10-I24A _Fm	gataatttatttttggctgataataattacatt
LEF-10-I24A _Rm	aatgtaattattatcagccaaaaataaattatc
LEF-10-I29A _Fm	atagataataattacgctattttaaatgtgttc
LEF-10-I29A _Rm	gaacacatttaaaatagcgtaattattatcta
LEF-10-V33A _Fm	tacattattttaaatgctttcgaccaagaaacc
LEF-10-V33A _Rm	ggtttcttggtcgaaagcatttaaaataatgta
LEF-10-C1F	agcctctagaacgaacgtatggttc
LEF-10-C3R	tagagaattccgtggacgcgttactttg
Del-test-F	attgaaccctccatcgtggtag
hsp104-test-R	ggaacaagtgacaaaggaacg
URA3-test-R	gaaaagctgtggtatggtgcac
DelHSP104-F	ggcaaaggggcgcaaacttatgcaacctgccagattattatataaggcgagcagattgtactgagagt gcacc
DelHSP104-R	caatttccatactgtcctcattatcgtcatcacctaacgtgtcagccttagttttgctggccgcatcttctc
LEF10-F (y)	ccgccaccgcggatggctagcacgaacgtatggttcgcgac
LEF10-R (y)	gagttcttctcctttgctagccgtggacgcgttactttgc
Sup35-F	ccgccaccgcggatggctagctcggattcaaaccaaggcaa

Supplementary Table 4 (count.)

Primer Name	Sequences (5'-3')
Sup35MC-F	ccgccaccgcggatggctagcatgtctttgaacgactttcaaaagc
Sup35-R	gagttcttctcctttgctagcctcggcaattttaacaattttacc
LEF10-TEST-F	aacgtatggttcgcgacgga
8XHIS-TEST-R	gtgatggtgatggtgatggtggtg
P(lef10)-LEF10-F	agagagatctggagtgcaacgtgatgcttc
P(lef10)-LEF10-R	attactcgagcgtggacgcgttactttgc
Lef10p-F	tatatagttgctgatgcaacgtgatgcttcgag
Lef10-R	cccggattaactccgttacgtggacgcgttact
pTGFP-R	atcagcaactatatattgatag
pTGFP-F	cggagttaatccgggacct
P(lef10)-LEF-10-GFP-HIS-F	agagagatctggagtgcaacgtgatgcttc
P(lef10)-LEF-10-GFP-HIS-R	taatgaattcttagtgatggtgatggtgatggtggtgcttgtacagctcgtccatgccg
GFP-TEST-F	tctcggcatggacgagctgtacaag
mCh-F	agtaacgcgtccacgggatccgactacaaagacgatgacgacaagg
mCh-R	gtggtggtggtggtgctcgagcttgtacagctcgtccatgcc
Lef-10-Muts-F	ctgtacaagggcggcggatccacgaacgtatggttcgcgac
Lef-10-Muts-R	cagctgtgcggccgcaagcttttacgtggacgcgttactttgc
